# A single application of fertilizer can affect semi-natural grassland vegetation over half a century

**DOI:** 10.1371/journal.pone.0275808

**Published:** 2022-11-30

**Authors:** Michio Tsutsumi, Syuntaro Hiradate, Masashi Yokogawa, Eri Yamakita, Masahito Inoue, Yoshitaka Takahashi

**Affiliations:** 1 Western Region Agricultural Research Center (Kinki, Chugoku and Shikoku Regions), National Agriculture and Food Research Organization, Shimane, Japan; 2 Faculty of Agriculture, Kyushu University, Fukuoka, Japan; 3 Osaka Museum of Natural History, Osaka, Japan; 4 The Shimane Nature Museum of Mt. Sanbe, Shimane, Japan; 5 Japan Grassland Conservation Network, Shimane, Japan; Feroze Gandhi Degree College, INDIA

## Abstract

Restoration of species-rich semi-natural grassland requires not only a seed source but also appropriate soil properties. In Europe, approximately 10 years are required for the properties of fertilized soils to reach suitable conditions and be considered successfully restored. However, restoration may require additional time in Japan because heavier precipitation causes leaching of basic cations from soils, resulting in soil acidification; volcanic ejecta also forms active Al and Fe hydroxides with high phosphate sorption. Within this context, we aimed to answer the following questions: i) whether and how the impacts of fertilization remain in the soil properties after half a century in Japan; and ii) how fertilization affects the restoration of semi-natural grasslands in Japan. We investigated the vegetation and soil properties of a *Zoysia japonica* pasture improved half a century ago with a single application of fertilizer and an adjacent semi-natural grassland (native pasture) in Japan, and found the following: (1) the two pastures had similar dominance of *Z*. *japonica*, but differed in the species composition; (2) the improved pasture exhibited lower species richness than the native pasture; (3) soil nutrients, including N, P, K, Mg, and Ca, were higher in the improved pasture than in the native pasture; and (4) many chemical properties of the soils were associated with species composition; namely, the vegetation on nutrient-rich soil had more alien species and fewer native species. We conclude that a single dose of fertilization can affect soil properties in semi-natural grasslands over half a century in Japan, leading to species loss and changing the species composition. We suggest that fertilized soils under grazing in Japan may require more than half a century to restore the nutrients to suitable levels for the establishment of a species-diverse grassland.

## Introduction

Grasslands are an important land cover with world-wide distribution, and these grass-dominated communities can occur both naturally and artificially [1]. Semi-natural grasslands maintained by human activities can also have diverse plants and animals, similar to natural grasslands. During the past century or earlier, the area of semi-natural grasslands has decreased because of land-use changes in many countries [1], including Japan [2, 3]. Consequently, many grassland plants and animals are endangered [1].

Numerous studies have been conducted on the restoration of semi-natural grasslands [4–8]. Restoration of species-rich semi-natural grassland requires not only a seed source for the grassland plants but also soil with favorable properties [9]. Low levels of extractable P, exchangeable Ca, and low pH are necessary for the successful restoration of heathlands or semi-natural grasslands in England [10]. Typical vegetation of semi-natural grassland in Japan is found only on soils with low levels of available P (<200 mg P_2_O_5_ kg^−1^ dry soil, Bray II) and low pH (H_2_O) (<5.7); alien species are seldom found under these conditions [11]. Similar results were obtained in Germany [12], where soil pH, P, and Mg are drivers of species composition and richness in semi-natural grasslands. However, a recent study stated that N was more important than P and K as a driver of species loss [13]. Therefore, eutrophication is a major driver of species loss in semi-natural grasslands.

Most arable soils in England are predicted to require at least 12 years before nutrient levels become suitable for the establishment of a heathland sward [10] based on experimental data [14]. Several methods to strip the soil of nutrients and promote acidification, including burning, cutting/mowing, topsoil stripping, cropping, and grazing, are available [15]. When these techniques are used, only several years may be required to restore the properties of fertilized soils to the condition before fertilization in England [10]. Although more than 50 years are required to restore semi-natural grassland vegetation on ex-arable fields in Sweden, young ex-arable fields (<10 years) and semi-natural grasslands have similar soil properties, including exchangeable P and pH [7].

The Japanese Archipelago lies in the northeast tip of the Asian Monsoon Zone and has heavier precipitation than Europe [16]. The heavier precipitation causes leaching of basic cations such as Ca^2+^, K^+^, and Mg^2+^ from the soils, resulting in soil acidification. In addition, the availability of soil P is very low in the natural Japanese ecosystem because volcanic ejecta, which covers wide areas of the archipelago, forms active Al and Fe hydroxides with high ability for phosphate sorption [17]. Therefore, the restoration period for limed and P-implemented soils in Japan is likely different from European soils. Although it was suggested that fertilization on grassland affected the vegetation over approximately 40 years in Japan [18], it has never been assessed the long-term effects of fertilization on soil properties.

We hypothesized that fertilized soils in Japan require more time (several decades or more) to restore species-rich semi-natural grasslands. Within this context we aimed to answer the following questions: i) whether and how the impacts of fertilization remain in the soil properties after half a century in Japan; and ii) how fertilization affects the restoration of semi-natural grasslands in Japan.

## Materials and methods

### Study site

The study site was located at Urumi, Chibu, Shimane, Japan (36.018° N, 133.014°E) at an altitude of 280–290 m in Chiburi Island (S1 and S2 Figs). This island belongs to the Oki Archipelago lying in the Sea of Japan. The area is within the temperate monsoon climate region. The annual average temperature is 14.9°C, and the annual average precipitation is 1589.3 mm (data are from Ama on Nakanoshima Island, near Chiburi Island [19]).

Semi-natural grasslands are distributed over the island. They were managed by cattle and horse grazing from April to June and from September to November, and by mowing in summer in the 1970s. In recent years, they are grazed only by cattle from May to November, continually. In 1970, part of the semi-natural grassland dominated by *Zoysia japonica* was improved without plowing [20]. The grassland was fertilized with 1,000 kg ha^−1^ of calcium carbonate, 500 kg ha^−1^ of fused magnesium phosphate, and 500 kg ha^−1^ of compound fertilizer (7% N, 12% P_2_O_5_, and 7% K_2_O). Introduced species were *Dactylis glomerata* (orchardgrass), *Lolium arundinaceum* (tall fescue), *Agrostis gigantea* (redtop), and *Arrhenatherum elatius* (tall oatgrass). Seeds were sown at rates of 20 kg ha^−1^ for each *D*. *glomerata*, *L*. *arundinaceum*, and *A*. *gigantea*, and 15 kg ha^−1^ for *A*. *elatius*. *Trifolium repens* (white clover) was also introduced, but the sowing quantity was unknown. All of these are exotic species. There was no fence between the improved pasture and the adjacent semi-natural grassland (hereafter, native pasture). After the improvement, the improved pasture was used together with the native pasture. Additional fertilizer was not applied.

### Data during 1972–1981

Vegetation data from each pasture during 1972–1981 were obtained as follows. Every July from 1972 to 1981, a vegetation survey was carried out in the improved pasture [20]. A systematic sampling design was used, with a 12.5 × 12 m grid resolution and 1 × 1 m quadrats. Twenty fixed quadrats were used for every survey. In each quadrat, all vascular plant species, coverage (%), and length were recorded. Plant cover and community height were also assessed. In the same period, a vegetation survey in the native pasture was also conducted [21] using the same design as the survey of the improved pasture [20], except for the grid resolution (8 × 25 m) and the number of quadrats (12). Some of the data for both the improved and native pastures were lost [22], including the raw data for 1979–1981. Species cover (%) data in each quadrat from 1972 to 1978 were classified into Penfound and Howard coverage classes [23]. However, full species lists and their mean covers (%) were available for 1972–1981 [22].

### Vegetation survey in 2019

On July 22 and 23, 2019, we conducted vegetation surveys in both the improved and native pastures. We employed the same design as the survey used for the improved pasture during 1972–1981 [20]. To precisely compare the vegetation in the two pastures, we also used 12.5 × 12 m grid resolution and 20 quadrats in the native pasture.

### Soil sampling and analysis in 2019

Soil sampling was conducted in both the improved and native pastures at the same time as the vegetation survey. Soil samples were collected from each quadrat used for the vegetation survey for analysis of chemical properties. The surface soil layer (0–5 cm) was sampled five times from each quadrat using a soil corer (5.0 cm height, 100 mL volume) and composited into one 500-mL sample. A soil profile survey was also carried out in the two pastures. The plot of the soil profile survey was selected to represent the typical vegetation in each of the improved and native pastures. For each soil horizon of the soil profiles, the soil mechanical impedance (soil compactness) was determined with a compactness tester (compact model Yamanaka, Fujiwara Scientific Company Co., Ltd., Tokyo, Japan). All the collected soil samples were air-dried, sieved through a 2-mm mesh, and subjected to further chemical analyses.

The values of soil pH in H_2_O (pH(H_2_O)) were determined using a standard pH meter with a glass electrode for a soil:water mixture of 1:2.5. The total C and N contents were determined by a combustion method. Cation exchange capacity (CEC) was determined by the Schollenberger method [24]. To determine exchangeable cations (Ca^2+^, Mg^2+^, K^+^, and Na^+^), the soil sample was extracted with ammonium acetate (pH 7.0) using the Schollenberger method, and this extract was analyzed with atomic absorption spectrophotometry. Soil NO_3_-N and NH_4_-N were extracted with 10% KCl solution with a soil:solution ratio of 1:10 (w:v) for 60 min. The NO_3_-N and NH_4_-N concentrations in the extracts were determined with naphthylethylenediamine absorptiometry and indophenol method, respectively. Hot-water-extractable N was determined by extracting N from a soil sample with boiling water using a soil:water ratio of 1:10 (w:v) for 60 min; extracted N was acid digested with concentrated H_2_SO_4_ at 200°C for 20 min and to 400°C for 5 min, followed by NH_4_-N determination using distillation method (Kjeldahl method).

Two methods were used to determine plant-available P in soil samples: the Troug method and Bray II method. In the Troug method, a 1 g portion of an air-dried soil sample was extracted with 200 mL of 1 M H_2_SO_4_ in 0.3% (NH_4_)_2_SO_4_ solution (pH 3.0) with shaking for 30 min. In the Bray II method, a 1 g portion of an air-dried soil sample was extracted with 20 mL of 0.03 M NH_4_F in 0.1 M HCl with hand shaking for 1 min. The inorganic phosphate concentrations in the extracts were determined with the molybdenum blue method [25]. Phosphate absorption coefficient (PAC) was determined as follows: a 10 g portion of a soil sample was reacted with 20 mL of 2.5% (NH_4_)_2_HPO_4_ solution (pH 7.0, 13,440 mg P_2_O_5_ L^−1^) for 24 h at room temperature, and the phosphate concentration in the supernatant was determined by the molybdenum yellow method [26].

### Data analysis

We performed statistical analysis using software R version 4.1.2 [27], including the R packages simpleboot [28], boot [29], vegan [30], and labdsv [31].

A nonparametric bootstrap resampling approach [32] was used to determine whether there was a significant difference between parameters from the two pastures. Bootstrap bias-corrected accelerated 95%, 99%, and 99.9% confidence intervals based on 10,000 random simulations were used to determine significance. When the 95% interval did not overlap between parameters, for example, we considered that the difference was statistically significant at the α = 0.05 level.

We used the Bray-Curtis index to quantify dissimilarity between species composition in the two pastures. The index between each quadrat in each year was calculated, and the effect of pasture on species composition was statistically tested using permutational multivariate analysis of variance (PERMANOVA [33]). Penfound and Howard’s coverage classes were used to indicate the abundance of species. The Bray-Curtis index takes a number between 0 and 1. If 0, the two quadrats share all the same species with the same coverage classes; if 1, they do not share any species.

We calculated the indicator value, IndVal [34], for each species to detect the indicator species in each pasture in 2019. In addition, a non-metric multi-dimensional scaling (NMDS) was performed to detect relationships between species composition and soil chemical properties in each quadrat in 2019. For these analyses, coverage (%) was used to indicate the abundance of species.

## Results and discussion

### Vegetation

**Fig 1:**
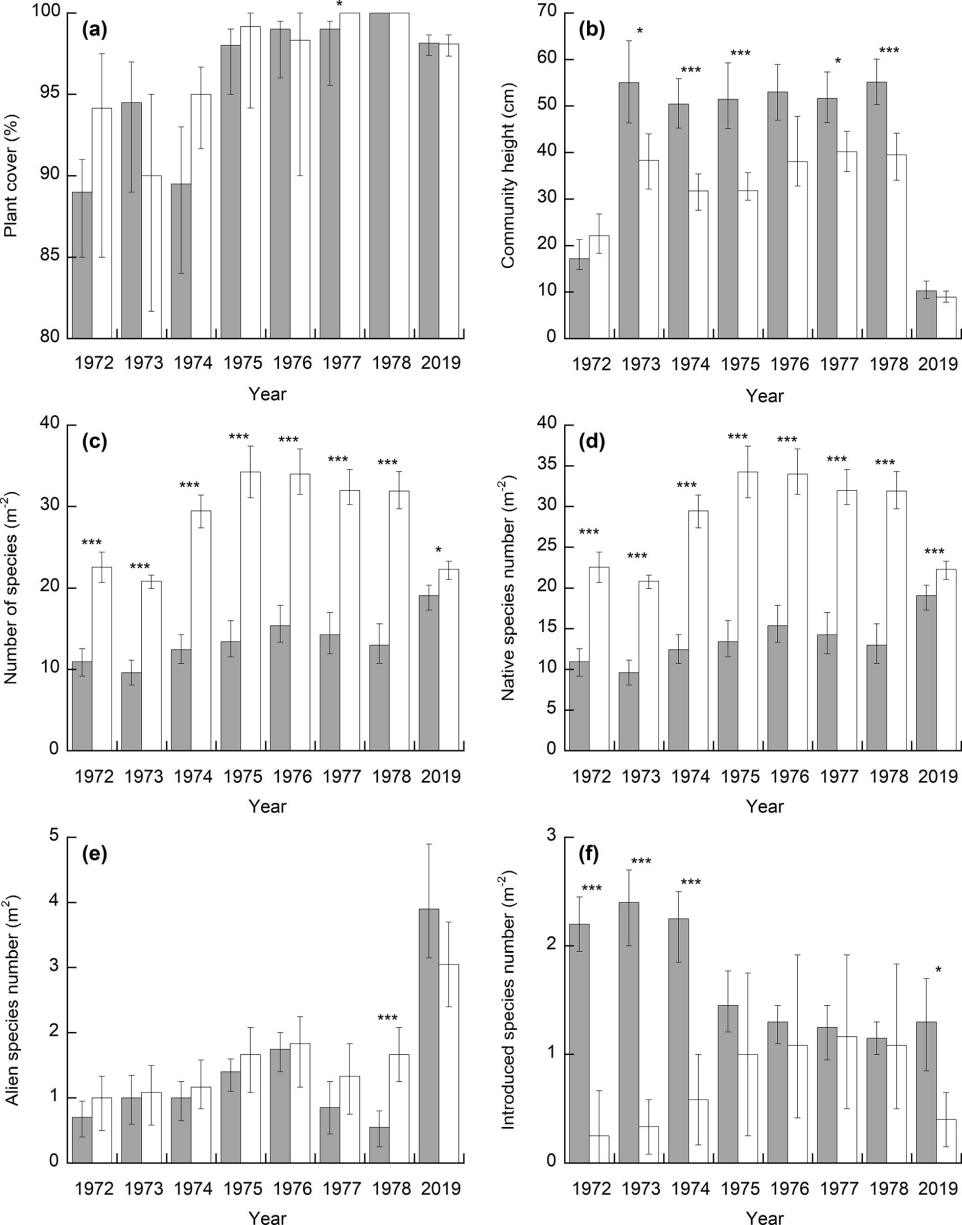
Profile of vegetation in the two pastures. Mean (a) plant cover, (b) community height, and the numbers of (c) all species, (d) native species, (e) alien species other than introduced species, and (f) introduced species per quadrat (m^2^) in the improved (gray) and native (white) pastures during 1972–1978 and 2019. Error bars indicate 95% bias-corrected accelerated confidence intervals. *p < 0.05, **p < 0.01, and ***p < 0.001.

In 1972, 2 years after the improvement, the total cover of the introduced grass species in the improved pasture was 4.9%, although one of the introduced species, *T*. *repens*, was dominant during 1972–1975 (S1 Table). This result indicates that it failed to establish pasture dominated by the sown grasses. During 1976–1981, the most dominant species in the improved pasture was *Artemisia indica* var. *maximowiczii*, a well-known typical field weed [35]. Because the alien weed *Vulpia myuros* became dominant after *A*. *indica* var. *maximowiczii* during 1980 and 1981, the vegetation during this period was similar to ex-arable land [36, 37]. In 2019, *Z*. *japonica*, which is a typical grass in semi-natural grassland managed by grazing [38], was dominant in the improved pasture, and the three introduced species—*T*. *repens*, *L*. *arundinaceum*, and *A*. *gigantea*—were present with 7.2, 4.5, and 3.4% cover, respectively (S1 and S2 Tables). *Z*. *japonica*, *Miscanthus sinensis*, and *Imperata cylindrica* var. *koenigii*, typical grasses in semi-natural grasslands [38], were dominant in the native pasture during 1972–1981. In 2019, *Z*. *japonica* was the dominant species with 62.8% cover, which was not significantly different from the improved pasture (54.6%). The number of all species and native species per quadrat in the native pasture was consistently higher than in the improved pasture during 1972–1978 and 2019 (Fig 1). The number of alien species per quadrat was not different between the two pastures except in 1978. In 2019, both the pastures had similar vegetation in terms of dominant species, its dominance, plant cover, and community height, but differed in species richness.

**Fig 2:**
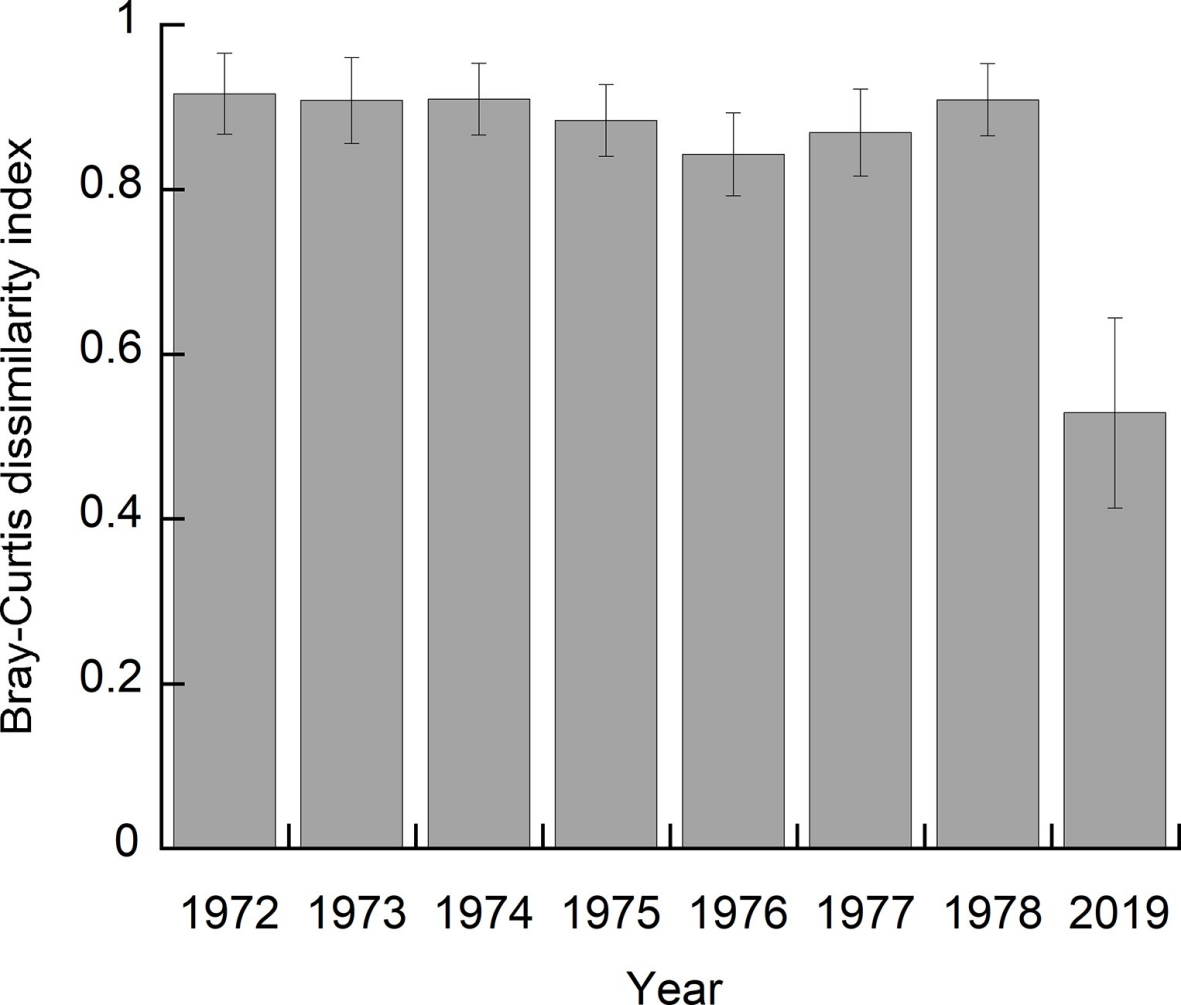
Dissimilarity between species composition in the two pastures. Mean of the Bray-Curtis dissimilarity index between the quadrats in the improved and native pastures during 1972–1978 and 2019. Error bars indicate standard deviation. The effects of pasture on species composition are statistically significant for all years (*p* < 0.001).

The mean Bray-Curtis dissimilarity index between the quadrats in the two pastures during 1972–1978 ranged from 0.843–0.916 (Fig 2), indicating that the species composition was significantly different between the pastures. In 2019, the dissimilarity index was 0.529, which was lower than in the 1970s, but the effect of the pasture on species composition was statistically significant during both periods.

Seven species were detected as indicators for improved pasture in 2019, including two introduced species (*T*. *repens* and *L*. *arundinaceum*), two alien species (*Poa pratensis* subsp. *pratensis* and *Rumex obtusifolius*), and three native species (*Oxalis corniculate*, *Aster microcephalus* var. *ovatusand*, and *Digitaria ciliaris*) (Table 1). *R*. *obtusifolius* and *D*. *ciliaris*, known as typical pasture weeds [39], were not found in the native pasture in any year (S1 Table). Indicators for native pasture in 2019 were *M*. *sinensis*, *Lysimachia japonica* f. *subsessilis*, *Paspalum thunbergia*, *Luzula capitata*, *Viola grypoceras* var. *grypoceras*, *Imperata cylindrica* var. *koenigii*, *Adenophora triphylla* var. *japonica*, *Rosa luciae*, *Dianthus superbus* var. l*ongicalycinus*, and *Phyllanthus lepidocarpus* (Table 1), all of which are native and typical species in semi-natural grasslands in Japan [22]. *V*. *grypoceras* var. *grypoceras*, *R*. *luciae*, and *P*. *lepidocarpus* were never or seldom recorded in the improved pasture (S1 Table). *Adenophora triphylla* var. *japonica* and *Dianthus superbus* var. *longicalycinus* were also found in the improved pasture during 1972–1981 but not in 2019.

**Table 1:** Indicator species for the two pastures in 2019.

Pasture	Indicator species	IndVal^a^	*p*	Frequency^b^		Mean cover (%)	
				Improved	Native	Improved	Native
Improved	*Oxalis corniculata*	0.7574	0.021	19	18	7.3	1.9
	*Trifolium repens*	0.5315	0.021	13	6	7.2	1.6
	*Lolium arundinaceum*	0.4402	0.004	9	2	4.5	0.1
	*Poa pratensis* subsp. *Pratensis*	0.4385	0.013	9	2	1.9	<0.1
	*Aster microcephalus* var. *ovatus*	0.3857	0.044	9	3	0.6	0.1
	*Digitaria ciliaris*	0.3500	0.007	7	0	0.2	0.0
	*Rumex obtusifolius*	0.3000	0.026	6	0	0.4	0.0
Native	*Miscanthus sinensis*	0.8889	0.001	5	19	0.3	4.7
	*Lysimachia japonica* f. *subsessilis*	0.7763	0.001	9	20	0.4	1.5
	*Paspalum thunbergii*	0.7050	0.014	16	20	3.7	8.9
	*Luzula capitata*	0.6923	0.001	6	18	0.2	0.5
	*Viola grypoceras* var. *grypoceras*	0.6910	0.001	1	14	<0.1	1.9
	*Imperata cylindrica* var. *koenigii*	0.6519	0.023	12	20	2.8	5.2
	*Adenophora triphylla* var. *japonica*	0.6000	0.002	0	12	0.0	0.8
	*Rosa luciae*	0.4424	0.009	2	9	0.1	5.8
	*Dianthus superbus* var. *longicalycinus*	0.4000	0.001	0	8	0.0	0.3
	*Phyllanthus lepidocarpus*	0.3000	0.024	0	6	0.0	0.2

^a^An indicator value.

^b^Per 20 quadrats.s

In a United States grassland, 10 years of N addition to the grassland decreased the plant diversity, and the grassland did not recover the same diversity during the following 20 years [40]. Our studies obtained similar results only after a single application of fertilizer (Fig 1). Although the dominant species was the same, with similar percent cover, the species composition in the native and improved pastures differed even half a century after the improvement (Figs 1 and 2). The same results were observed in a pasture approximately 40 years after improvement by plowing and fertilization in the Kyushu region of Japan [18]. These results suggest that fertilization can have a long-term effect on species composition over several decades or more.

### Soil properties

Soil profiles from both the improved and native pastures indicated weak soil structures with a similar horizon composition, boundary, soil color, field texture, and consistency (Table 2). Roots were more abundant in the A horizon of the soil profile in the improved pasture than in the native pasture. Both soil profiles were acidic (pH(H_2_O) < 5.2) with low levels of exchangeable cations, and had high phosphate adsorption ability, indicated by high PAC between 13.0 and 16.3 g P_2_O_5_ kg^−1^ (Table 3). A PAC value higher than 15 g P_2_O_5_ kg^−1^ has been used to characterize volcanic ash soils with a high ability to adsorb phosphate, which could induce phosphorus deficiency for upland plants in Japan [41]. Although both profiles were not classified as Andosols because the thickness criteria were not satisfied, their ability to suppress phosphorus efficacy for plants was high, and phosphorus is a key factor controlling plant growth. From those characteristics, both soils were classified as Cambisols according to the World Reference Base for soil resources [42] and Inceptisols according to the United States Soil Taxonomy [43]. Table 3 clearly shows that available phosphates, as determined by Bray II P and Troug P, were higher through the soil profile of the improved pasture than in the native pasture.

**Table 2:** Soil profiles in the two pastures.

Pasture	Horizon	Depth (cm)	Boundary	Color	Field texture	Structure			Consistence			Root		Soil type
						Size^a^	Grade	Type^b^	Stickiness^c^	Plasticity^d^	Compactness	Size^e^	Abundance^f^	
Improved	A	0–5	Wavy gradual	7.5YR 3/3	CL	VF, F, M	Weak	G	SS	SP	Loose	VF, F	C	Cambisol [42]
						M, C	Weak	SAB				M	VF	Inceptisol [43]
	Bw1	5–15	Wavy gradual	7.5YR 4/3	CL	F, M, C	Weak	SAB	SS	SP	Loose	VF, F	F	
												M	VF	
	Bw2	15–23	Wavy gradual	7.5YR 4/3	LiC	F, M, C	Weak	SAB	S	P	Loose	VF, F	F	
	Bw3	23–31	Wavy gradual	7.5YR 4/3	LiC	F, M, C	Weak	SAB	S	P	Medium	VF, F	VF	
	Bw4	31–50	Wavy gradual	7.5YR 4/3	LiC	F, M, C	Weak	SAB	S	P	Medium	VF, F	VF	
	Bw5	50–72	Wavy gradual	7.5YR 4/3	LiC	F, M, C	Weak	SAB	S	VP	Medium		N	
Native	A	0–8	Wavy gradual	7.5YR 3/3	LiC	VF, F	Weak	G	SS	P	Medium	VF, F	F	Cambisol [42]
						M, C	Weak	SAB				M	VF	Inceptisol [43]
	Bw1	8–24	Wavy gradual	7.5YR 4/3	LiC	F	Weak	SAB	S	VP	Medium	VF, F	F	
	Bw2	24–43		7.5YR 4/3	LiC	M, C	Weak	SAB	S	VP	Medium	VF, F	VF	

Both soils are derived from basaltic volcanic ejecta.

^a^VF: very fine, F: fine, M:medium, C: coarse.

^b^SAB: subangular brocky, G: granular.

^c^SS: slightly sticky, S: sticky.

^d^SP: slightly plastic, P: plastic, VP: very plastic.

^e^VF: very fine, F: fine, M: medium.

^f^N: none, VF: very few, F: few, C: common.

**Table 3:** Soil chemical properties of horizons in the two pastures in 2019.

			Total content					Exchangeable cations							Available phosphate	
Pasture	Horizon	pH(H_2_O)	C	N	C/N	PAC	CEC	Ca	Mg	K	Na	NO_3_-N	NH_4_-N	HN	Bray II P	Troug P
			(%)			(g P_2_O_5_ kg^-1^)	(cmol_c_ kg^-1^)					(mg kg^-1^)			(mg P_2_O_5_ kg^-1^)	
Improved	A	4.84	8.38	0.85	9.9	13.3	38.5	6.1	5.7	2.1	0.3	100	151	452	273	86
	Bw1	4.68	3.24	0.34	9.5	15.0	30.7	1.8	1.1	0.6	0.3	15	55	79	188	41
	Bw2	4.72	2.52	0.25	10.1	14.7	28.5	1.6	0.9	0.6	0.3	6	64	55	223	53
	Bw3	4.74	2.10	0.24	8.8	16.3	26.1	1.4	0.7	0.6	0.2	2	34	47	219	57
	Bw4	4.75	1.92	0.21	9.0	14.7	25.6	1.3	0.6	0.6	0.2	3	36	41	214	58
	Bw5	4.82	1.67	0.19	8.8	13.9	23.2	1.2	0.4	0.6	0.2	1	27	31	148	39
Native	A	5.15	6.56	0.59	11.1	13.0	30.9	5.1	4.5	0.9	0.5	11	158	290	148	45
	Bw1	5.20	2.51	0.27	9.2	14.0	25.0	2.7	2.3	0.2	0.5	5	58	62	43	9
	Bw2	5.19	1.70	0.18	9.3	13.7	21.8	2.1	1.5	0.2	0.5	2	49	31	24	5

Abbreviations: PAC—phosphate absorption coefficient, CEC—Cation exchange capacity. HN—Hot-water-extractable N. All data were calculated on the basis of oven-dried (105°C) soil.

The chemical properties of the surface soils between 0 and 5 cm depth are listed in Table 4 (see also S3 Table). In the improved pasture, NO_3_-N, NH_4_-N, and hot-water-extractable N (NH_4_-N plus labile precursors of NH_4_-N and NO_3_-N) were significantly higher than in the native pasture. These increased N concentrations were likely derived from the fertilized N in 1970 and seemed to cause higher total N content and lower C/N ratio without influencing total C content. Available phosphates (Bray II P and Troug P) and exchangeable Ca and K were also significantly higher in the improved pasture than in the native pasture, and likely originated from fertilizers applied in 1970. These results imply that applied nutrients, such as P, N, K, and Ca, can remain as available forms for plants in surface soils for a long time (e.g., half a century). These nutrients are actively recycled and regenerated in the soil-plant ecosystem.

**Table 4:** Chemical properties of surface (0–5 cm depth) soil in the two pastures in 2019.

			Total content					Exchangeable cations							Available phosphate	
Pasture		pH(H_2_O)	C	N	C/N	PAC	CEC	Ca	Mg	K	Na	NO_3_-N	NH_4_-N	HN	Bray II P	Troug P
			(%)			(g P_2_O_5_ kg^-1^)	(cmol_c_ kg^-1^)					(mg kg^-1^)			(mg P_2_O_5_ kg^-1^)	
Improved	Mean	5.07	7.93	0.76	10.4	15.2	40.1	7.4	5.5	2.3	0.4	41	142	397	299	77
	Upper limit^a^	5.14	8.34	0.80	10.7	15.7	41.8	8.3	6.0	2.5	0.5	56	161	438	355	90
	Lower limit^b^	5.00	7.52	0.72	10.2	14.8	38.7	6.7	5.0	2.0	0.4	30	122	362	258	66
Native	Mean	5.08	7.59	0.65	11.7	13.4	34.4	5.2	4.5	1.4	0.4	12	109	320	127	41
	Upper limit	5.12	8.02	0.68	11.9	13.9	35.3	5.5	4.7	1.6	0.5	14	115	346	152	48
	Lower limit	5.04	7.23	0.62	11.5	13.0	33.6	4.9	4.2	1.3	0.4	10	104	301	109	36
Statistical significance		NS	NS	**	***	***	***	***	**	***	NS	***	*	*	***	***

Abbreviations: PAC—phosphate absorption coefficient, CEC—Cation exchange capacity. HN—Hot-water-extractable N.

^a^Upper limit of 95% confidence interval.

^b^Lower limit of 95% confidence interval.

*p < 0.05

**p < 0.01

***p < 0.001; NS: not significant. All data were calculated on the basis of oven-dried (105°C) soil.

### Relationship between vegetation and soil properties

**Fig 3:**
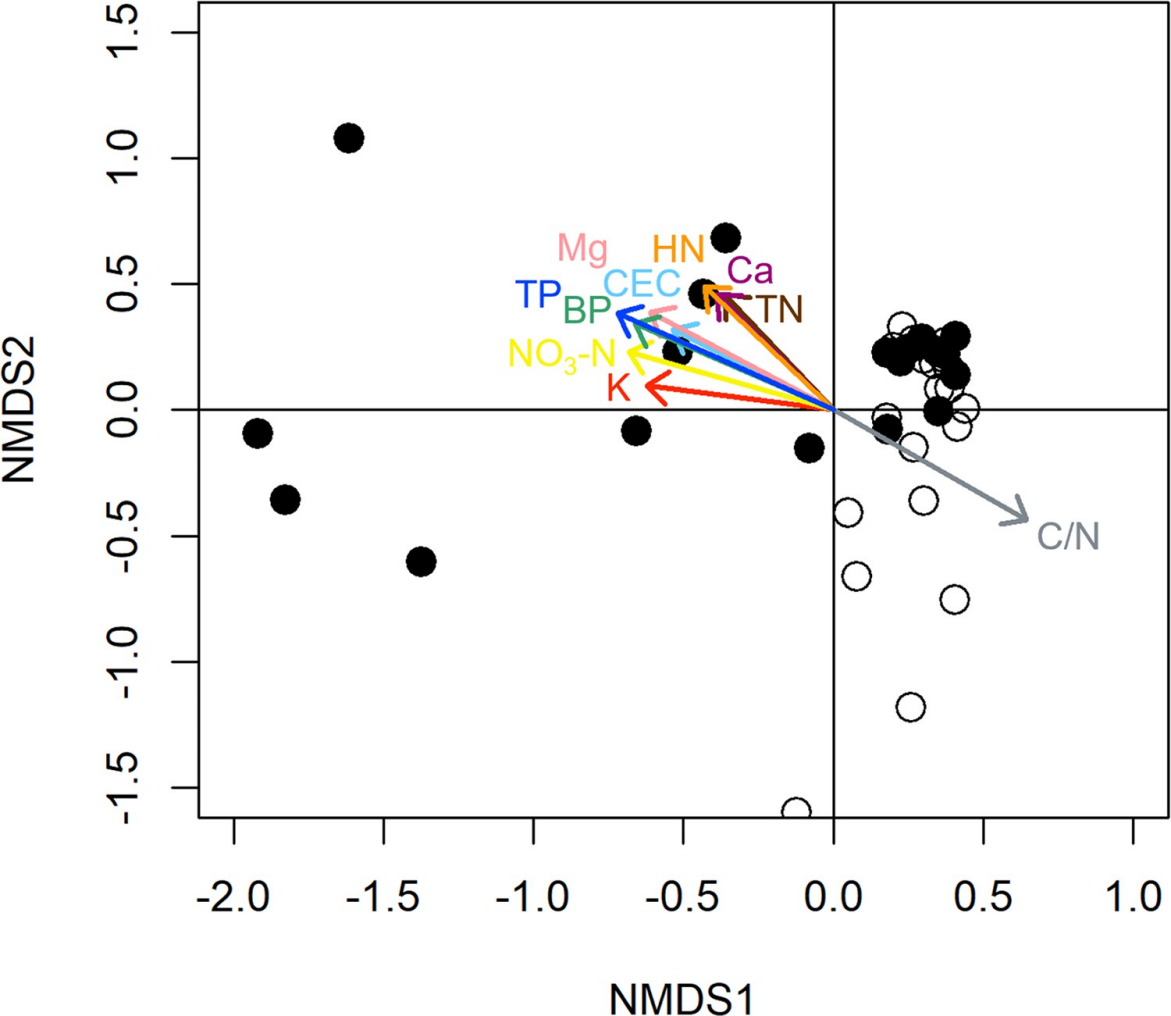
Relationship between species composition in the two pastures and soil chemical properties in 2019. Non-metric multi-dimensional scaling (NMDS) ordination of plant species composition in the improved (filled circle) and native (open circle) pastures in 2019. The vectors represent the significant (*p* < 0.05) chemical properties associated with the species composition. Abbreviations: NMDS1 and NMDS2, axes 1 and 2 for the NMDS, respectively; TN, total N, HN, hot-water-extractable N; BP, Bray II P; TP, Troug P; CEC, cation exchange capacity; Ca, exchangeable Ca; Mg, exchangeable Mg; and K, exchangeable K.

The NMDS plot (Fig 3) revealed that species composition in the two pastures in 2019 differed (Fig 2), while part of quadrats in the two pastures had similar species composition. The following 10 chemical properties were significantly associated with the species composition: total N, C/N, CEC, exchangeable Ca, exchangeable Mg, exchangeable K, NO_3_-N, hot-water-extractable N, Bray II P, and Troug P (Fig 3). Soil pH (H_2_O) was not a significant parameter, presumably because there was no difference between the pastures. All of these significant vectors, except C/N, took negative values on the NMDS axis 1 (NMDS1) and positive values on axis 2 (NMDS2). NMDS1 was negatively correlated with the number of alien species (excluding introduced species) and introduced species, and NMDS2 was negatively correlated with the number of native species (and with all species) (Table 5). The numbers of alien species and native species were not correlated. All the quadrats that took negative NMDS1 and positive NMDS2 values belonged to the improved pasture, and most of the quadrats that took positive NMDS1 and negative NMDS2 values belonged to the native pasture. These results indicate that quadrats on nutrient-rich soil had more alien species and fewer native species, and vice versa. The nutrient-rich soil resulted from the dose of fertilizer in 1970 (Tables 3 and 4). Therefore, our results suggest that a single dose of fertilizer affects the species composition in semi-natural grassland even after half a century or more.

**Table 5:** Pearson correlation matrix for the NMDS axes shown in Fig 3 and species composition.

	NMDS1^a^	NMDS2^a^	Native Species^b^	Alien Species^b^^,^^c^	Introduced Species^b^
NMDS2	0.000				
Native species	0.289	-0.479**			
Alien species	-0.589***	0.264	-0.266		
Introduced Species	-0.617***	0.221	-0.347*	0.331*	
All species^b^	-0.184	-0.320*	0.797***	0.314*	0.072

^a^See Fig 3.

^b^Number per quadrat.

^c^Other than introduced species.

*p < 0.05

**p < 0.01

***p < 0.001.

A previous study assessed the influence of soil chemical parameters (pH, P, K, and Mg) on species composition in semi-natural grasslands, and found that P, Mg, and pH together explained 17% of the variation in species composition [12]. In our analysis, the 10 chemical properties, including not only P and Mg but also K, were significant parameters associated with species composition (Fig 3). Responses of plants to the level of chemical properties in soil vary by species [44], which may cause the differences in species composition. Although responses of wild plant species to edaphic mineral variations were recently assessed, the available data are still limited [44]. A database of plant species responses to edaphic mineral variations will accelerate the understanding of how mineral content drives species composition.

## Conclusions

We investigated the vegetation and soil properties of a *Z*. *japonica* pasture that was improved half a century ago with a single dose of fertilizer and an adjacent semi-natural grassland in Japan. We found the following: (1) the two pastures were dominated by *Z*. *japonica* with similar percent cover, but differed in the species composition; (2) the improved pasture exhibited lower species richness than the native pasture; (3) soil nutrients, including N, P, K, Mg, and Ca, were higher in the improved pasture than in the native pasture; and (4) many soil chemical properties were associated with the species composition, namely the vegetation on nutrient-rich soil had more alien species and fewer native species. Therefore, we conclude that fertilization, even a single dose, can affect soil properties in semi-natural grasslands after half a century in Japan, leading to species loss and affecting species composition. In addition, we suggest that fertilized soil under grazing in Japan may require more than half a century for the nutrients to be restored to suitable levels for the establishment of a species-diverse semi-natural grassland.

## Supporting information

S1 Fig The location of Chiburi Island. The study site was located in Chiburi Island, belonging to the Oki Archipelago. The original maps were created by Esri Japan Corporation, Tokyo, Japan (available at https://www.esrij.com/products/japan-shp/).
(TIFF)

S2 Fig The study site. The improved and native pastures were located within and outside of the blue polygon, respectively. The sky-blue and red squares indicated the plots for survey at the improved and native pastures, respectively. The aerial image was copyrighted by Nariyasu Watanabe (Western Region Agricultural Research Center, NARO).
(TIFF)

S1 Table Plant species composition in the two pastures. Plant species recorded in the two pastures during 1972–1981 and 2019, and their mean covers (%).
(XLSX)

S2 Table Vegetation data in the two pastures in 2019. Plant species recorded in each quadrat in 2019, and their covers (%).
(XLSX)

S3 Table Chemical properties of surface (0–5 cm depth) soil in each quadrat in 2019. (XLSX)
