# Supplementary figures and images for: A single application of fertilizer can affect semi-natural grassland vegetation over half a century
Source: PLoS One. 2022 Nov 30;17(11):e0275808. doi: 10.1371/journal.pone.0275808 (PMC9710762; doi:10.1371/journal.pone.0275808)

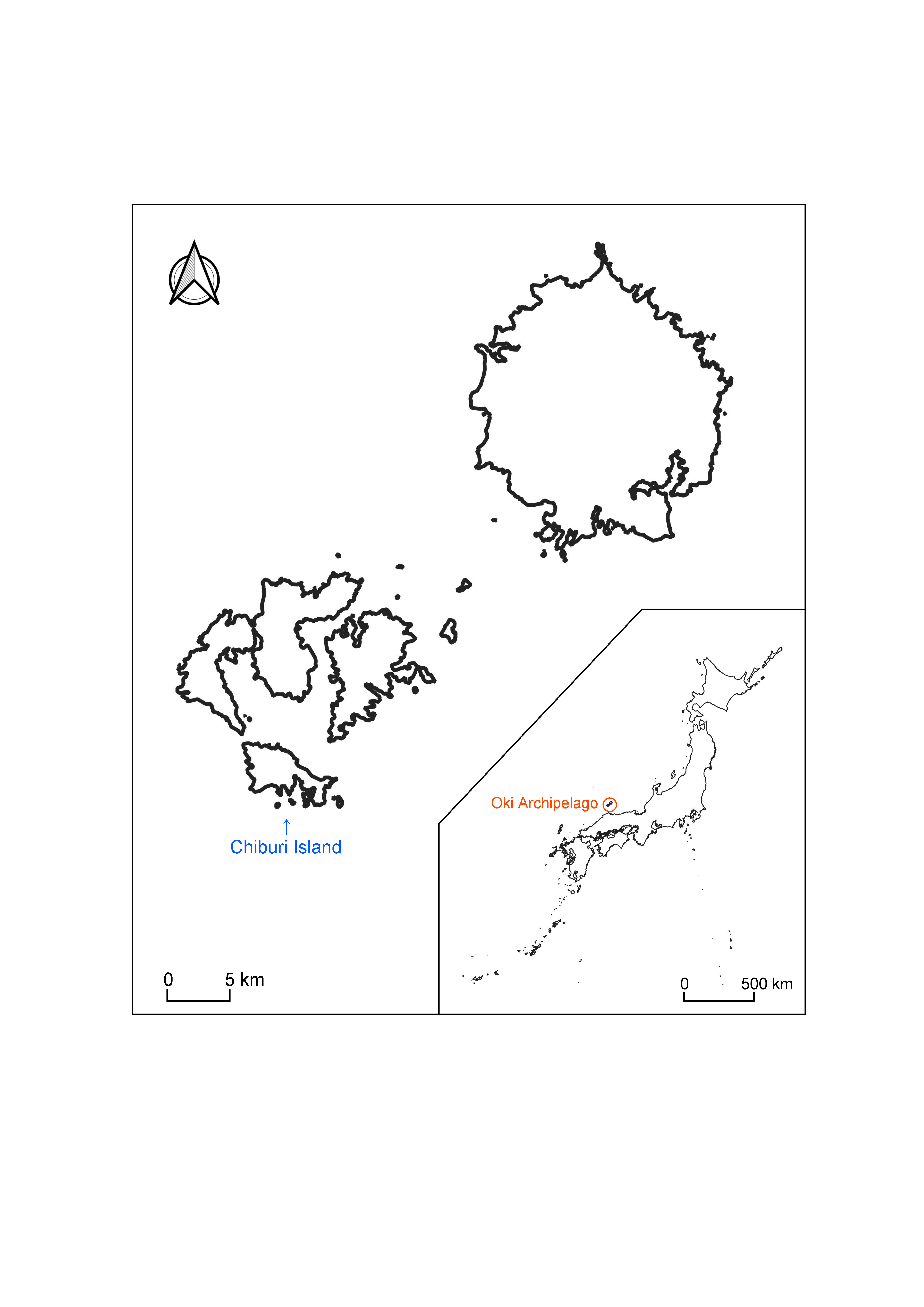

Supplement: S1 Fig — The study site was located in Chiburi Island, belonging to the Oki Archipelago. The original maps were created by Esri Japan Corporation, Tokyo, Japan (available at https://www.esrij.com/products/japan-shp/). (TIFF) [file pone.0275808.s001.tiff]

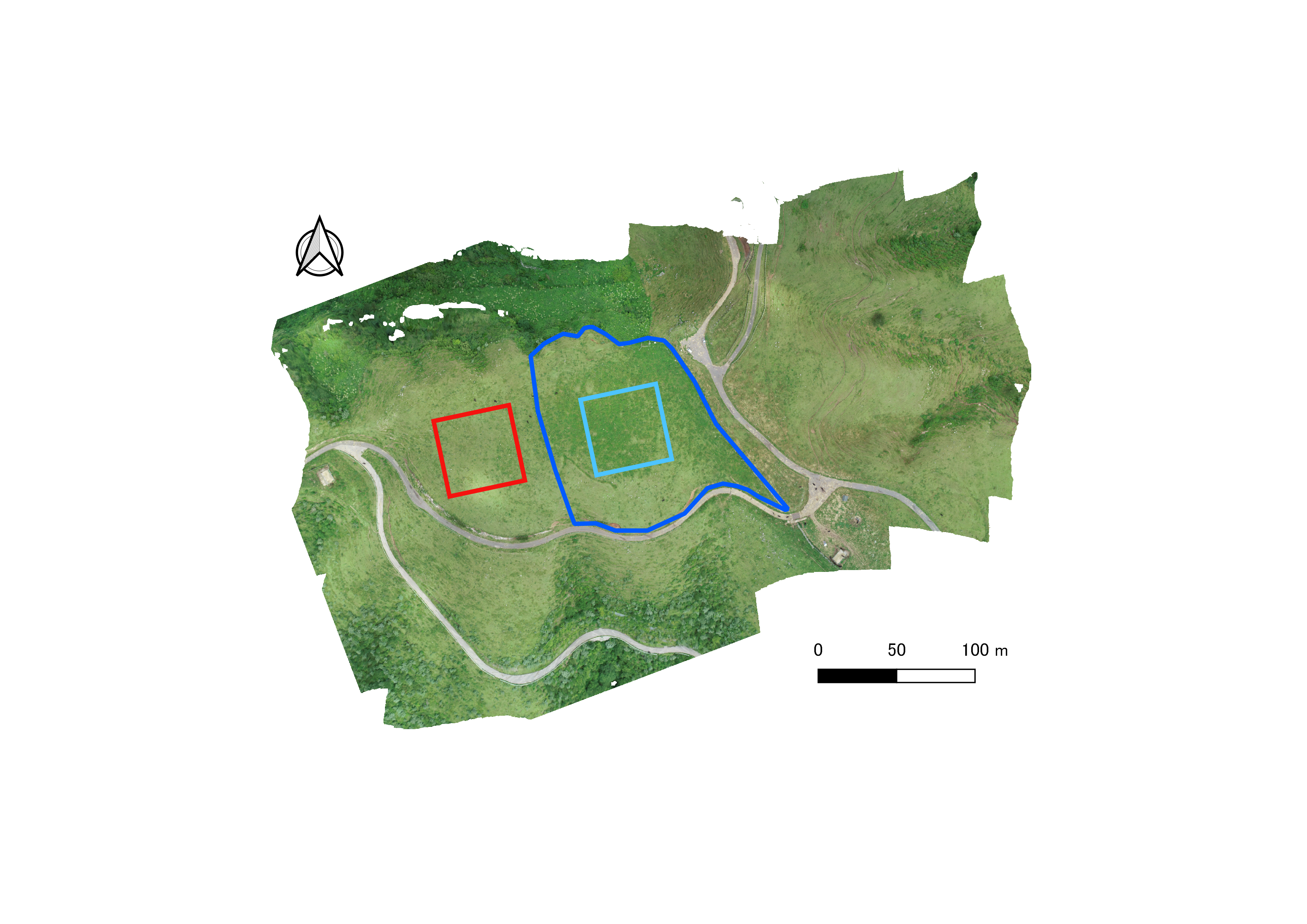

Supplement: S2 Fig — The improved and native pastures were located within and outside of the blue polygon, respectively. The sky-blue and red squares indicated the plots for survey at the improved and native pastures, respectively. The aerial image was copyrighted by Nariyasu Watanabe (Western Region Agricultural Research Center, NARO). (TIFF) [file pone.0275808.s002.tiff]
